# The Influence of Probiotics Consumption on Management of Prediabetic State: A Systematic Review of Clinical Trials

**DOI:** 10.1155/2022/5963679

**Published:** 2022-09-12

**Authors:** Shabnam Zeighamy Alamdary, Roghayeh Afifirad, Sajjad Asgharzadeh, Parisa Asadollahi, Marzie Mahdizade Ari, Shirin Dashtibin, Mohamad Sabaghan, Mohammad Reza Shokouhamiri, Roya Ghanavati, Atieh Darbandi

**Affiliations:** ^1^Department of Bacteriology, Faculty of Medical Science, Tarbiat Modares University, Tehran, Iran; ^2^Department of Microbiology, School of Medicine, Tehran University of Medical Sciences, Tehran, Iran; ^3^Department of Microbiology, School of Medicine, Iran University of Medical Sciences, Tehran, Iran; ^4^Microbial Biotechnology Research Centre, Iran University of Medical Sciences, Tehran, Iran; ^5^Microbiology Department, Faculty of Medicine, Ilam University of Medical Sciences, Ilam, Iran; ^6^Behbahan Faculty of Medical Sciences, Behbahan, Iran; ^7^Faculty of Paramedicine, Golestan University of Medical Sciences, Gorgan, Iran

## Abstract

Prediabetes consists of the intermediary stage between normal glucose regulation and overt diabetes mellitus and develops when blood glucose levels are higher than normal but not high enough to confirm a type 2 diabetes mellitus diagnosis (T2DM). Recent evidence suggests that probiotics could be promising approaches to improve this state. In this study, we performed a systematic review to compile the results of clinical trials investigating the effects of pro-/pre-/synbiotics on prediabetes subjects from 2010 to 2020. The article search was carried out in Medline, Embase, Scopus, Web of Science, The Cochrane Library, Clinical trials.gov, ProQuest, Open Grey, and Google Scholar. Search filters were developed using 2 parameters: “prestate diabetes” and “probiotics.” Of the 418 studies that were screened, 15 original articles reached the inclusion criteria. Pooling data from these trials showed positive and significant effects of probiotics in the reduction of hyperglycemia, insulin concentration levels, lipid profile, and BMI (Body mass index). Administration of probiotics may provide beneficial and healthful effects in the clinical management of patients with prediabetes and metabolic syndrome. Different probiotics compositions have shown beneficial and noticeable effects on glucose homeostasis, lipid profiles, BMI, and inflammatory markers in subjects with prediabetes, metabolic syndrome, and healthy individuals and could be advantageous in recomposing the gut microbiota back into the normal state during the prediabetic state.

## 1. Introduction

Prediabetes (preDM) is a high-risk condition in which glycemic levels pass the normal range but not the diabetes cut-offs. It is qualified by impaired glucose metabolism and dysregulation of insulin secretion, demonstrated as raised fasting glucose, impaired glucose tolerance, or a combination of both [1]. The incidence of prediabetes is increasing worldwide and type 2 diabetes mellitus (T2DM) is highly likely to develop in individuals with this condition. However, not all preDM subjects develop overt T2DM. Changes in life style, diet, and medications can prevent or delay the development of preDM to T2DM [2]. One study has demonstrated that early intervention in prediabetes can reduce the risk of developing type 2 diabetes by 58% [3]. However, in clinical settings, hypoglycemic medication is only rarely used as an effective approach to control preDM outcomes in high-risk populations. Therefore, finding nonhypoglycemic agents based on lifestyle modification in preventing the conversion of prediabetes to diabetes will be beneficial and significant. The composition and activity of the gut microbiota codevelop since birth and it has been shown that the proportion and diversity of the gut microbiome is drastically modified (also known as intestinal dysbiosis) in the early prediabetes period [4], obesity [5], and T2DM [6,7]. This alteration is thought to cause an increase in the intestinal permeability and endotoxemia, which in turn sets off a chronic low-grade inflammatory response and the emergence of insulin resistance. Numerous strategies have been used to control glucose levels and regulate imbalances in the gut microbiome such as the use of antibiotics, synbiotics, postbiotics, and probiotic [8–10].

FAO/WHO (Food and Agriculture Organization/World Health Organization) has outlined probiotics as live bacteria that when administered in adequate amounts could have a health benefit on the host [11]. They may help in the maintenance of healthy gut which can act as effective supplements in insulin resistance therapies, exert antidiabetic effects, and improve glucose homeostasis [12, 13]. Probiotics, such as the bacteria of the genera *Bifidobacterium* and *Lactobacillus*, are relatively well-tolerated by the host and have been suggested to moderately improve glycemic control, retard the onset of hyperglycemia, hyperinsulinemia, dyslipidemia, and oxidative stress, and improve the overall intestinal health [14, 15]. Further, prebiotics are determined as specific indigestible components such as inulin and oligofructose/galactose complex which specifically provide the condition for the growth of probiotics and are appealing potential therapeutic options for preDM and T2D by regulating blood glucose, insulin sensitivity, and lipid metabolism. Indeed, modulation of intestinal microbiota by pro-/pre-/synbiotics is an interesting approach in reversing changes in the gut microbiota along the progression of metabolic syndrome in preDM [16–18].

Hence, this systematic review was conducted on published randomized controlled trials (RCTs) on prediabetic patients among which the effects of probiotic supplementations were investigated on modulation of the gut microbiota and control of obesity and metabolic disorders like diabetes.

## 2. Materials and Methods

This systematic review is performed in agreement with the requirements outlined in Preferred Reporting Items for Systematic Reviews and Meta-Analysis Statement, 2020 (PRISMA) [19]. Our systematic review has been registered in PROSPERO (international prospective register of systematic reviews): CRD42021236421 (https://www.crd.york.ac.uk/PROSPERO/display_record.php?Recordid=236421). A literature search was conducted using the PICO concept (Participants, Intervention, Control, Outcome) [20]. The participants were overweight and obese patients, healthy and prediabetes adults, adolescents with prediabetes, and elderly patients with metabolic syndrome; the intervention was different strains of probiotics alone or in combination with prebiotics, prebiotic agent alone, and yogurt; the control included patients receiving placebo, no treatment; the primary outcomes were glycemic parameters (FBG, GA, GLP-1, and PYY), insulin factors (HOMA-IR, HOMA-B, QUICKI, and HbA1c), lipid profiles (TC, LDL-c, HDL-c, triglycerides, and VLDL), and BMI. The secondary outcomes variables were gut microbiota composition and inflammatory markers indexes.

### 2.1. Electronic Search

To identify relevant articles, a structured search strategy was carried out through the following databases: Medline, Embase, Scopus, Web of Science, The Cochrane Library, Clinical trials.gov, and ProQuest. Also, a search was conducted for grey literature in OpenGrey and Google Scholar. Reference lists of all related publications and systematic reviews were also checked to identify any potentially relevant articles. These issues were done to overcome any defects of electronic databases and search engines. Initial search was based on keywords derived from our research questions. The keywords used were “pre-state diabetes” and “probiotics” which were also searched in combination using the Boolean operators to improve the results. Also, the search strategy was adapted to the particularities of each database. Whenever possible search for synonyms or similar terms was done before every keyword. The search strategy is described in Supplementary Data 1.

### 2.2. Data Collection and Analysis

**Figure 1:**
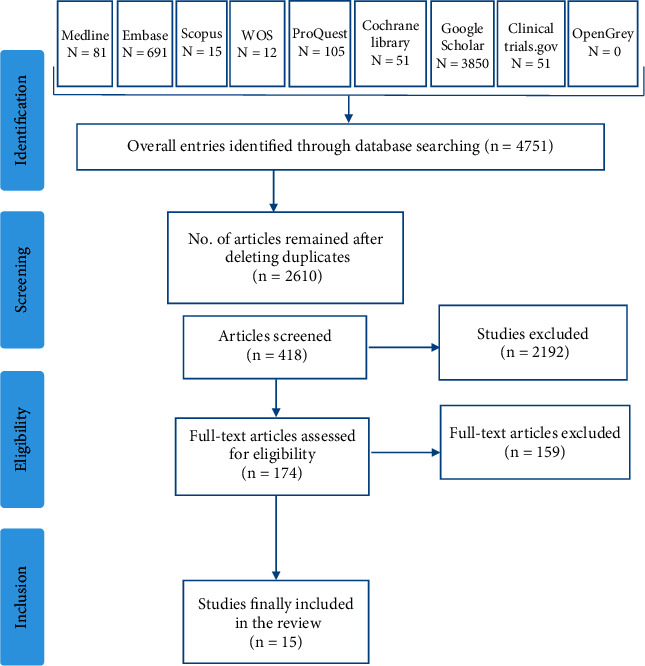
PRISMA flow diagram of the systematic review literature search results.

Figure 1 summarizes the articles explored. The literature search, data extraction, and quality assessment were independently done by two of the authors. Any discrepancies in the results were resolved by consulting a third author. Publications extracted from more than one database were included only once. The initial selection of the articles was based on the analysis of title and abstract and finally reading the main text to choose them based on the eligibility criteria. A study was qualified for inclusion if the following criteria were met: in [1] the study was a RCT; in [2] it was conducted on prestate diabetic patients who had been given probiotics; [3] was published in English language from 2010 to 2020. The exclusion criteria were non-RCT publications; animal experiments; congress papers; abstracts without full text; studies with no clear information; reviews. The data extracted from each study included first author's name, year of publication, country of the study, sample size, mean weight of the study subjects, study design, participants characteristics, types of interventions (whether probiotic, prebiotic, or synbiotic), probiotics strains, probiotics dose, controls used, duration of the therapy, and main outcomes. The qualitative variables were analyzed using the independent two-sample *t*-test. SPSS software version 18.0 (IBM, NY, USA) was used for the statistical analysis, and the statistical significances were achieved when ^*∗*^*P* < 0.05.

### 2.3. Quality Assessment

The quality of the references was assessed using the Joanna Briggs Institute (JBI, 2014) [21]. A score range of 0 to 13 points was given to each involved publication. Every eligible article was rated as “yes,” “no,” “unclear,” or “not applicable.” Finally, the papers with high quality were included in the current study (Supplementary Data 2).

## 3. Results

In an initial search, 4751 articles were found and by reviewing the titles and abstracts, 2192 articles were excluded for different reasons (such as duplicate studies, nonrelevant topics, and non-English papers) and 174 articles were retained for detailed full-text evaluation. The following reasons led to the exclusion of 159 articles: they were not original papers (books, editorials, comments, and reviews) and animal model studies. Finally, 15 articles describing the pro-/pre-/synbiotic efficacy on prediabetes treatment were selected for further analysis (Table 1).

**Figure 2:**
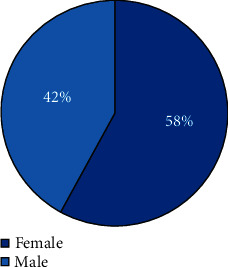
Prediabetes frequency among male and female patients.

Most of the studies had been performed in Iran (7 out of 15 studies and 660 out of 1295 patients). Out of the 15 included studies, only 8 had reported the gender of the subjects, which included 58% females and 42% males (Figure 2). The age groups were reported in 13 studies (14–58 years old). In all the 15 trials, blood specimens were collected at the beginning and end of the study. Stool specimens were also assessed in 4 studies.

**Figure 3:**
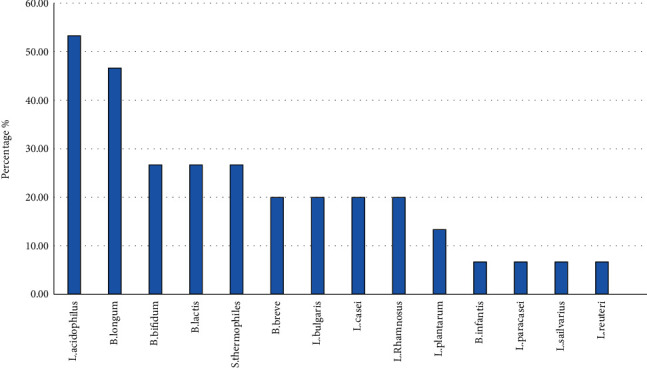
The frequency of probiotic species used to treat preDM patients in different studies.

Among the 15 clinical trials, 9 used a combination of multistrain probiotic bacteria, among which one used 8 probiotic types, two used 7 types, and 4 trials used 4 combination types of probiotics. In 3 trials, single probiotic strains were used. In one trial, low-fat conventional and fortified yogurt were introduced as probiotic products. Also, 2 trials evaluated the effects of the prebiotics xylooligosaccharide (XOS) and MSPrebiotic® in experimental groups and one trial used probiotic capsules consisting of 7 types of probiotic strains. Seven out of the 15 studies explored the effects of a multispecies synbiotic supplement consisting of probiotic bacteria with a prebiotic agent (Table 1). A total of 15 different probiotic species were administered at dose ranges of 1 × 10^7^ to 1 × 10^11^ colony forming units (CFU), with an optimum dose of 7 × 10^10^ CFU. *L*. *acidophilu*s (56.25%) and *B. Longum* (50%) were the most common probiotics used by different studies (Figure 3).

In a trial by Tay et al. [22] it was reported that there was an improvement in the mental health and social functioning scores of prediabetes in the *Lacticaseibacillus rhamnosus* (*L*. *rhamnosus*) probiotic supplementation group, and no improvement was seen in the placebo subjects. Similarly, participants in this study reported an overall improvement in their general health including physical functioning, role physical, bodily pain, vitality, and emotional role after the intervention by *Lacticaseibacillus rhamnosus* (*L. rhamnosus*) [22].

On the other hand, Sartang et al. [23] showed an inverted relationship between changes in the 25-hydroxyvitamin D (25(OH)D) concentration and the body fat mass (FM) by yogurt consumption. It was reported that a significant increase (from the baseline) was seen in the total 25(OH)D level among the group using fortified synbiotic yogurt (FSY) (*P* < 0.001) compared to the low-fat conventional yogurt consumption group (*P* < 0.001). The percentage of individuals with serum 25(OH)D concentrations of <75 nmol/L reduced from 79.6% at the baseline to 15.9% at the end of the study in the FSY consumption group but was not modified significantly in the low-fat conventional yogurt group (74.5% to 65.1%) [23].

### 3.1. Biochemical Parameters

Assessing glycemic (biochemical) parameters included evaluation of fasting plasma glucose or fasting blood sugar (FPG or FBS) concentrations, serum insulin levels (INS), fasting plasma insulin (FPI), homoeostatic model assessment for insulin resistance (HOMA-IR), insulin sensitivity (measured by ISI-M), fasting insulin levels (FIL), homoeostatic model assessment for *β* cell function (HOMA-B), quantitative insulin sensitivity checks index (QUICKI), *C*-peptide, and glycated hemoglobin (HbA1c) levels.

### 3.2. Glycemic Parameters

**Figure 4:**
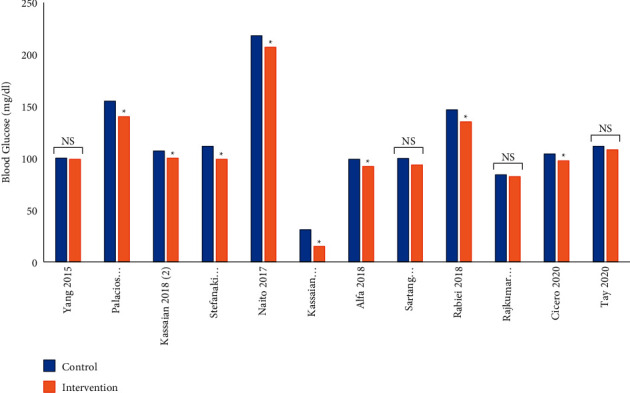
Serum glucose levels in control (baseline) and intervention (pro-/pre-/synbiotic) groups.

According to Figure 4, 12 studies investigated the effect of probiotic, prebiotic, or synbiotic supplementations on FPG, FBS, or serum glucose concentrations. Four of the 12 studies reported nonsignificant reductions in FBG or serum glucose levels in groups receiving pro-/pre-/synbiotic in comparison to the control groups. In Naito et al. [24] trial, fasting and postload plasma glucose (PG) levels did not differ between the groups at any visits. However, 1 hr postload PG levels were significantly attenuated at 8 weeks compared to the baseline in the probiotic *Lactobacillus casei* strain *Shirota* (*LcS*) group (*P*=0.036) and Glycoalbumin (GA) levels significantly decreased at 8 weeks compared to the baseline in the probiotic *LcS* group (*P*=0.002) [24]. Kassaian et al. found a significant decreasing trend for the incidence of hyperglycemia (FPG ≥100) in the probiotic and synbiotic subjects (*P*=0.01 and *P*=0.005, respectively) [25]. Also, in Placios et al. study, despite the lack of a significant difference of fasting plasma glucose (FPG) between the probiotic and placebo groups, a decrease in the FPG level was seen in probiotic group receiving or taking metformin [26]. In addition, Rebiei et al. revealed that synbiotic consumption significantly increased glucagon-like peptide-1 (GLP-1) and PYY (Peptide YY) levels in metabolic syndrome patients. Although GLP-1 increased in both groups after 12 weeks, the rate of increase in the synbiotic group was remarkably higher than that in the placebo group [27].

### 3.3. Insulin Factors

**Figure 5:**
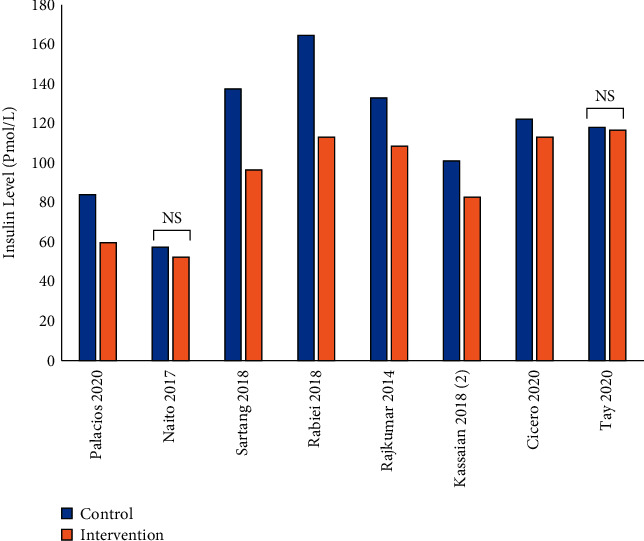
Serum insulin levels in control (baseline) and intervention (pro-/pre-/synbiotics) groups.

According to the results of the trials mentioned in this review, changes in insulin factors are variable. As shown in Figure 5, among the 15 clinical trials, 8 trials reported changes in insulin levels [22–24, 26–30]. In 6 trials, insulin levels were decreased noticeably in probiotic and synbiotic groups [23, 26–30]. In Naito et al. study, there was no notable difference in the indices for insulin sensitivity and insulin secretion in the *LcS* probiotic group compared to the baseline [24]. In addition, Tay et al. [22] reported that insulin levels remained unchanged after the intervention by *L. rhamnosus* [22]. Among the 15 clinical trials, 9 trials examined the effect of pro-/pre-/synbiotic on homoeostatic model assessment for HOMA-IR [23, 24, 26–32]. The results of 6/9 studies indicated that, in participants taking intervention, a significant decrease in HOMA-IR was found from the baseline to the end point of the studies [23, 26, 27, 29–31]. However, HOMA-IR changes were not significant in the intervention groups of the other 3 studies [24, 28, 32]. Based on Alfa et al. results, insulin resistance (IR) was calculated by three methods: the lipoprotein insulin resistance (LP-IR), HOMA-IR, and QUICKI-IR. The LP-IR was not significantly different between MSPrebiotic® and placebo groups over time in either elderly (ELD) or mid-age (MID) healthy adults. However, HOMA-IR and QUICKI-IR values revealed remarkable improvement over time in IR for individuals on MSPrebiotic® compared to the placebo group (*P*=0.009 and 0.004, respectively) [31].

Two trials by Naito et al. and Kassaian et al. examined homoeostatic model assessment for *β* cell function (HOMA-B). HOMA-B levels remained unchanged during probiotic or synbiotic administration in these trials [24, 29]. On the other hand, Kassaian et al. and Alfa et al. reported a significant increase in the QUICKI among the synbiotic and prebiotic groups through the intervention period [29, 31]. Moreover, Sartang et al. reported that QUICKI was greater in the FSY group compared to the low-fat conventional yogurt (LFY) group and baseline [23]. Furthermore, 5 trials reported significant decreases in the HbA1c levels from the baseline following the probiotics and synbiotic supplementations, compared to the placebo groups [22, 24, 26, 29, 33].

### 3.4. Lipid Profiles

Total cholesterol (TC), low-density lipoprotein-cholesterol (LDL-c), high-density lipoprotein-cholesterol (HDL-c), triglycerides, and very low-density lipoprotein (VLDL) were considered as lipid profile indicators in the studies analyzed.

The activity of lipid indexes was evaluated in 9 studies (Table 2) [22–25, 27, 28, 30, 31, 34], among which 5 reported that the intake of pro/ pre/synbiotics is not related to any valid changes in the levels of LDL-c, TC, triglyceride (TG), and HDL-c [22, 25, 27, 31, 34]. Results from Sartang et al. showed an increase in the HDL-c level in the FSY group following the 10-week intervention and a decrease in the serum triglyceride level in both the FSY and LFY groups, with a greater effect in the FSY group (*P*=0.003) [23]. In a separate study, Alfa and colleagues reported a significant increase in the large VLDL and chylomicron particles with MSPrebiotic® supplementation (*P*=0.02). In this study, lipid profile, particle size profile, TG, and systemic cholesterol levels were largely unaffected following the consumption of MSPrebiotic® in ELD and MID adults. Naito et al. reported that TC (*P*=0.023), LDL-c (*P*=0.022), and non-HDL-C (*P*=0.008) levels were significantly lower in the *LcS* group compared to the placebo group following 8 weeks of intervention. Triacylglycerol (TAG) levels did not change during the trial in this study [31]. In Mahboobi et al. study, probiotic supplementation did not contribute to notable changes in the TC, LDL-C, HDL-C, TG/LDL, TG/HDL, and LDL-C/HDL-C ratios, after 8 weeks [34]. Kassaian et al. [29] showed that hypertriglyceridemia was considerably lower in both probiotic and synbiotic groups compared to the placebo group at 24 weeks of intervention (*P*=0.02). Moreover, the prevalence of low HDL-cholesterol was reduced only in probiotic group compared to the placebo group.

### 3.5. Effects of Probiotic, Prebiotic, and Synbiotic Supplementations on the Gut Microbiota in Healthy and PreDM Individuals

Four trials investigated the effects of probiotic, prebiotic, or synbiotic administration on the fecal microbiota among the preDM subjects [26, 32, 33, 35].

Yang et al. [32] found that 8-week XOS intervention had a clear influence on the gut microbiota in both healthy and preDM subjects, resulting in powerful shifts in the abundance of several bacterial taxa associated with preDM. Among these taxa, *Dialister* spp. and *Slackia* were proinflammatory markers and were greatly reduced by XOS. Yang et al. showed that XOS intervention significantly decreased (*P* ≤ 0.05) the load of the phylum Firmicutes in healthy subjects. Also, an increase in the *Streptococcus* and *Subdoligranulum* abundance in the placebo group was largely inhibited by XOS among healthy subjects. On the other hand, XOS (xylooligosaccharides) intervention significantly increased *Blautia hydrogenotrophica* abundance in the preDM subjects (*P* ≤ 0.05) [32].

Palacios et al. indicated that multistrain probiotic supplementation for 12 weeks increased the relative abundance of *Bifidobacterium breve, Akkermansia muciniphila,* and *Clostridium hathewayi (cluster XIVa)* and decreased the abundance of the proinflammatory bacteria *Prevotella copri*. No significant differences in the bacterial beta diversity at the species level were detected between the probiotic and placebo groups from the baseline at week 12 [26].

Kassaian et al. administrated probiotics and synbiotics for six months on the intestinal microbiome composition of prediabetes adults and found significant increase in the *Bacteroides fragilis* to *E. coli* abundance ratio (*P*=0.04). The proportion of *Cholestridium perfringens* (Firmicutes) to *Bacteroids fragilis* (Bacteroidetes) ratio was dramatically decreased in the probiotic supplementation group. Synbiotics had no significant effect on the composition or the relative proportions of the intestinal microflora [35].

In a pilot trial by Stefanaki et al., intestinal microbiome analysis showed statistically significant variations between the two groups of adolescent prediabetes; the probiotic intake group revealed significantly lower incidences of *Barnesiella* spp. (*P*=0.01), *Butyrivibrio crossotus* (*P*=0.01), *Faecalibacterium prausnitzii* (*P*=0.01), *Collinsella aerofaciens* (*P*=0.03), *Escherichia coli* (*P*=0.01), and *Akkermansia muciniphila* (*P*=0.03), compared to the control [33].

### 3.6. Inflammatory Markers

Inflammatory markers in some studies were assessed by the evaluation of IL-6, IL-1*β*, TNF*α*, CRP (C-reactive protein), and adipocytokines such as leptin, adiponectin, and pancreatic polypeptides (PP).

Yang et al. found that no marked XOS-related changes were shown in leptin, PP, or the inflammatory marker TNF*α* levels among the prediabetic patients [32]. Rabiei et al. also did not demonstrate a statistically significant reduction in the IL-6 and hs-CRP (high-sensitivity C-reactive protein**)** levels following synbiotic supplementation [27]. Rajkumar et al. showed that the serum concentrations of CRP, IL-1*β*, IL-6, and TNF-*α* were significantly (*P* < 0.05) reduced in the probiotic and synbiotic groups compared to the control group. Furthermore, reduction in the concentrations of these factors was more significant (*P* < 0.05) in the synbiotic group compared to the probiotic group among healthy volunteers [30]. Also, based on the results of the Alfa et al. study, supplementation with MS prebiotic had no effects on the IL-10 levels in the ELD or MID groups. Consumption of MS prebiotic for 3 months was not sufficient to reduce the elevated CRP and TNF-*α* levels in the ELD group [31]. Tay et al. [22] reported that no significant differences were observed in the concentrations of the liver enzymes aspartate transaminase (AST) and alanine aminotransferase (ALT), nor the levels of the inflammatory markers IL-6 and TNF-*α* following probiotic intervention among the diabetic patients [22]. Cicero et al. demonstrated in a 2-month treatment that patients who received synbiotic treatment experienced a statistically valid improvement in hs-CRP and TNF-alpha serum levels, compared to the baseline and the placebo group [28].

### 3.7. Body Weight and BMI Indexes

Among the 15 studies included, only 7 had reported the effects of pro-/pre-/synbiotics on weight changes and signs of the overall adiposity such as BMI [23–25, 27–30, 32]. In a study by Rabiei et al., synbiotic supplementation had a massive impact on the reduction of weight, BMI, and calorie intake at weeks 6 and 12 compared to the beginning of the study in synbiotic and placebo groups (*P* < 0.001) [27]. In addition, Cicero et al. showed significant improvements in the waist circumference (WC) and visceral adiposity index (VAI) among the treatment group who received synbiotics for 2 months compared to the control group [28].

Rajkumar et al. observed that, after 45 days of administration of probiotic, the BMI did not alter in the placebo and treatment groups, while it was significantly reduced (*P* < 0.05) in the synbiotic (probiotic plus fructooligosaccharides (FOS)) group, when compared to the baseline as well as the end point values of the control and probiotic groups [30]. Moreover, Sartang et al. from Iran determined that the daily consumption of FSY and LFY decreased mass (kg), BMI, waist circumference, body FM, and body fat percentage compared to the baseline. Reductions in waist circumference (*P*=0.002), body FM (*P*=0.023), and body fat percentage (*P*=0.028) were vaster in the FSY group compared to the LFY group [23]. In 2 studies, no significant change in the BMI was observed following probiotic consumption [29, 32]. On the other hand, Naito et al. study on the probiotic *Lactobacillus casei* showed that the body weight, BMI, and percentage of body fat markedly increased from the baseline at each visit after the start of the intervention [24].

In general, about 85% of the studies included in this review reported positive modulating effects for the pro-/pre-/synbiotics treatments on glucose levels, lipid profile, and intestinal microbial composition compared to the placebo groups. Even in studies where the effects of these supplementations were not significant, no adverse outcomes were reported.

### 3.8. Risk of Bias Included Studies

In this review, the majority of clinical trials showed low risk of bias “yes” in all questions (13 questions) except blinded outcomes assessors (Question 6). This item was “unclear” in 12 out of 15 studies. In Question 13, 4 studies were marked as “no” because the publications did not follow a standard guideline to perform the RCT designs and did not specify a method to conduct the study. The results of the evaluation of the bias risk are displayed in Supplementary Data 2.

## 4. Discussion

Prediabetes is determined as an intermediate step between normoglycemia and overt diabetes mellitus with impaired fasting glucose (IFG) and glucose tolerance (IGT), predisposing individuals at high risk of developing diabetes and its complications. In 2015, the International Diabetes Federation estimated that the worldwide incidence of IGT in adults was 415 million and was expected to reach 642 million by 2040 [36, 37]. In the current systematic review, we evaluated the effects of pro-/pre-/synbiotics on metabolic parameters, BMI, and gut microbiota composition in prediabetic patients, metabolic syndrome, and healthy individuals. Data analysis from 15 studies showed a significant and positive effect of pro-/pre-/synbiotics in the reduction of fasting plasma glucose, fasting insulin levels, total cholesterol, triglyceride, CRP, and HbA1c levels as well as beneficial modulations of the gut microbiome composition.

According to the results of the studies involved in present review, taking pro-/pre-/synbiotics had beneficial effects on lowering FPG or FBS levels in prediabetic patients. It seems that the effect of pro-/pre-/synbiotics on glucose metabolism is established through different mechanisms. Many studies have reported that growth or metabolic activity of some bacteria including *Lactobacillus* or *Bifidobacterium* spp. is stimulated by pro-/pre-/synbiotics substances and that these agents could increase the short-chain fatty acids (SCFA) levels in the colon [38–40]. In addition, primary bile acids can be converted into secondary bile acids by *Lactobacillus* and *Bifidobacterium* species to stimulate the production of GLP-1 from the intestinal L cells [41, 42]. Moreover, a combination of *Bifidobacterium* and *Lactobacillus* spp. can enhance glucose tolerance and boost SCFA and butyrate synthesis that stimulate the intestinal production of GLP-1 [43]. To confirm this pathway, Rebiei et al. revealed that synbiotic supplementation dramatically increased levels of GLP-1 and PYY in individuals with the metabolic syndrome [27]. Kassaian et al. for first time in Iran revealed synbiotic could improve hyperglycemia in 21.4% of the patients significantly [25]. On the other hand, Alfa et al. suggested that MSPrebiotic® might be a useful and efficient strategy in increasing the growth of Bifidobacteria and controlling blood glucose level, particulary in healthy elderly adults [31]. In healthy adults, this should not be surprising, given that the gluconeogenesis pathway will adapt the body by generating glucose to suppress hypoglycemia and glycemic level control [44].

On the other hand, previous articles have reported that there is a strong association between gut microbiota dysbiosis, which is characterized by an increase in the number of proinflammatory *Proteobacteria*, and the bacterial endotoxin production, which promotes the expression of Toll-like receptor 4 (TLR4) and inflammatory markers (e.g., TNF-*α*) and increase IR level, eventually leading to hyperglycemia and T2D [45]. And also, another theory is that Gram-negative bacteria LPSs bind to and activate the TLR4/CD14 complex, which activates proinflammatory markers and IR [46]. So, probiotics supplementation can decrease bacterial LPSs and proinflammatory cytokines, thus leading to advancement of insulin sensitivity [47]. Stefanaki et al. reported that the majority of Gram-negative bacteria (*Barnesiella* spp., *E. coli, Akkermancia muciniphila*) decreased following probiotic intervention; possibly the LPSs of these bacteria contributed to insulin resistance pathway [33, 48, 49]. According to some studies, the severity of the symptoms hypertension, dyslipidemia, and obesity are significantly correlated with insulin resistance and elevated CRP concentrations [50]. So, the ability of probiotics to reduce inflammation and hs-CRP can be a key indicator of their ability to reduce insulin resistance [51]. Also, insulin sensitivity is improved by supplementation of whey protein [52], calcium [53], vitamin D [54], and synbiotic [55] in prior studies. Along with these results Sartang et al. reported that the developments in HOMA-IR and QUICKI were detected by FSY consumption [23]. In context to the beneficial effects of pro-prebiotics on endotoxemia, inflammation, and IR, GLP-1 and GLP-2 have also been noticed to play an important role, and in a prebiotic diet on mice, markedly increased levels of circulating GLP-1 and GLP-2 have been discovered. So, intake of prebiotics and increased levels of GLP-1 and GLP-2 responses are linked to increased levels of beneficial gut bacteria, improved intestinal barrier health, and decreased levels of metabolic inflammation and endotoxemia [43, 48, 56]. So Rajkumar et al. defined that maybe a GLP-associated approach is involved in the promoting effect of *L. salivarius* plus FOS on IR and glucose tolerance [30]. Further, a previous meta-analysis reported that synbiotic intake can reduce HOMA-B in a statistically significant manner in diabetic participants [57]. However, Kassaian et al. showed HOMA-B remained unchanged during probiotic or synbiotic intervention [29]. Alfa et al. revealed that MSPrebiotic® significantly lessened blood glucose and insulin levels in the ELD individuals and reduced IR as measured by HOMA-IR and QUICKI-IR [31].

Emerging evidence showed that the consumption of pro/prebiotics can improve intestinal microbiota composition, resulting in increased production of SCFA and saccharolytic fermentation metabolites and improved intestinal barrier function [58, 59]. Based on studies on healthy individuals, XOS notably decreases the prevalence of bacteria associated with T2DM or obesity, including those from the phylum Firmicutes and the genera *Bacilli* and subdoligranulum [60, 61]. In addition, XOS may have an effective role in weight control and inhibit the trend of diabetes by inhibiting the growth of *Firmicutes* and increasing the *Oscillospira* abundance [62]. Palacios et al. detected increased level of *Bifidobacterium breve* and *Akkermansia muciniphila* as acetate-producing bacteria and a decline in the prevalence of the proinflammatory bacteria *Prevotella copri* in the probiotic consumption group [26]. It has been demonstrated that *A. muciniphila* can maintain bacterial homeostasis and restore the thickness of the intestinal mucus layer and impose antiobesity effects. These effects have been related to higher intestinal epithelial cell turnover and an attenuation in carbohydrate absorption [63, 64].

Mahboobi et al. revealed that the use of probiotic capsules on a daily basis had no major impacts on the blood lipids levels including TC, LDL-C, and HDL-C along with TG/LDL-C, TG/HDL-C, and LDL-C/HDL-C after two months [34]. On the other hand, Kassaian et al. found that the predominance of low HDL-cholesterol further developed through probiotic utilization [25]. Ataie-Jafari et al. showed that the daily utilization of 300 g probiotic-advanced yogurt decreased total and LDL cholesterol levels compared to the control subjects and *LcS* suppressed the increased blood cholesterol concentrations [65]. Although different probiotic strains and species have diverse modes of action that result in hypocholesterolemia, the following mechanisms have been hypothesized: (1) cholesterol absorption; (2) cholesterol binding/incorporation to cellular components, such as the cell surface or membrane; (3) bile acid deconjugation via bile-salt hydrolase; (4) inhibition of de novo synthesis of cholesterol via SCFAs; (5) ability to inhibit hypercholesterolaemia in obese prediabetic individuals by reducing dietary cholesterol absorption via binding to and/or assimilating sterols; (6) lowering the serum cholesterol concentrations by competing with cholesterol for intestinal absorption [66, 67].

Rideout et al. [33] found that MSPrebiotic® supplementation led to considerable increases in the abundance of total HDL particles in Western diet swine model, driven primarily by a rise in the small HDL subclass of particles [68]. Alfa et al., however, showed that MSPrebiotic® made no noticeable difference on the levels of total cholesterol, HDL, or LDL, in either MID or ELD healthy people [31]. In addition, Kassaian et al. demonstrated that synbiotic consumption for 6 months led to a decline in the serum triglyceride levels in prediabetic subjects [69]. Liong et al. used a synbiotic food containing *L. acidophilus*, FOS, inulin, and mannitol in hypercholesterolemic pigs and observed a decrease in serum triglycerides level after 8 weeks. The possible mechanisms by which synbiotics reduce triglycerides include lipolysis of triglycerides, inhibiting the NF-*κ*B pathway, regulating the gut microbiota-SCFA-hormone axis, and deconjugation of intestinal bile salts by bacterial bile-salt hydrolase [70].

Sartang et al. believed that an increase in calcium intake due to utilization of FSY may contribute to a reduction in body fat mass [23]. Besides, prior studies have reported that calcium-supplemented diets have an impact on FM loss [71, 72]. On the other hand, it has been reported that FSY intake markedly improves serum 25(OH)D concentrations. A reverse connection between changes in 25(OH)D concentrations and body FM were detected in Sartang study [23], which was in consistence with previous reports that documented the effects of vitamin D consumption on the reduction of the overall body mass, FM, FFM, and adipogenesis [73–75]. There are a variety of ways that probiotics or prebiotics may help reduce obesity and related metabolic disorders which include manipulation of gut microbiota by increasing SCFAs production in the large intestine, endotoxin reduction, preventing the expansion of visceral adipocytes, reducing appetite and inducing the feelings of satiety, which is linked to a plasma rise of the gut peptide concentrations (GLP-1 and PYY) [76].

In conclusion, this systematic review suggests that pro-/pre-/synbiotic supplementations have beneficial and helpful effects on metabolic parameters, microbiota composition, and obesity indexes in prediabetes individuals. This systematic review's notable strength is that, since 2010, a lot of studies have been assessed, with the majority of trials displaying a low risk of bias. Besides, the current study collected a broader range of biomarkers and outcomes such as different types of interventions (pro/pre and synbiotics), dosages, and duration of intervention. Nevertheless, several limitations should be noted in this systematic review: (a) Applying different methodology and protocols in the included studies such as utilizing a diverse array of probiotic strains, prebiotic agents, dosage of probiotics used, and the average age of trials participants may be the major cause for various impacts of probiotics on glycemic factors, insulin, and lipid profiles; (b) short intervention period in some studies may not be long enough to provide an significant impress on prediabetes individuals; (c) exclusion of unpublished trial data and non-English original articles in this study prevented us from obtaining absolute results. To substantiate these findings, additional human researches relating probiotics and prediabetes with no negative outcomes are required along with evaluation of further confounding variables and potential therapeutic agents. Furthermore, performing meta-analyses, which might make it easier to compare human study results fairly in the future, is required [76].

## Figures and Tables

**Table 1:** Summary of results of included studies.

First author, year	Country	Sample size T C	Mean age (SD)	Mean weight (SD)	Participants characteristics	Prebiotics	Probiotics	Probiotics dose (CFU)	Intervention	Control used and duration of therapy	Outcomes
Alfa, 2018	Canada	42T 42C	58.5	77.55	ELD (>70 years) and MID (30–50 years) adults	DRS	Not reported	Not reported	12 wk DRS 30 g/D	14 wk food grade corn starch 30 g/D	Dietary supplementation with prebiotics may be part of an effective strategy to reduce IR

Cicero, 2020	Italy	30T 30C	71.5	BMI (kg/m^2^) 27.35	Elderly patients with a diagnosis of MetS	Inulin + FOS	*L. acidophilus*	2 × 10^9^	2 mth o.d probiotic + (inulin + FOS + magnesium stearate) biphasic vial	2 mth o.d placebo (maltose + magnesium stearate) biphasic vial	Treatment with a synbiotic decreased MetS syndrome prevalence, several cardiovascular risk factors, and markers of insulin resistance in elderly patients
							*L. plantarum*				
							*L. reuteri*				

Kassaian, 2018	Iran	80T 40C	52.9	Not reported	Prediabetic individuals were recruited from the first relatives of type 2 diabetic patients	Inulin	*L. acidophilus*	1.5 × 10^9^	24 wk probiotic synbiotic 6 g/D sachet form	24 wk maltodextrin 6 g/D sachet form	Potential benefits of using probiotic and synbiotic to metabolic syndrome management in prediabetes patients
							*B. bifidum*				
							*B. lactis*				
							*B. longum*				

Kassaian, 2018	Iran	80T 40C	52.95 ± 6.3	78.4 ± 11	Prediabetic subjects	Inulin	*B. lactis*	1 × 10^9^	24 wk probiotic synbiotic 6 g/D powder	24 wk maltodextrin 6 g/D powder	Glycemic index improved by probiotics and synbiotic supplements in prediabetic individuals
							*B. bifidum*				
							*L. acidophilus*				
							*B. longum*				

Kassaian, 2019	Iran	80T 40C	52.95 ± 6.3	78.4 ± 11	Prediabetic subjects	Inulin	*B. lactis*	1.5 × 10^9^	6 mth probiotic synbiotic 6 g/D sachet form	6 mth maltodextrin 6 g/D sachet form	Probiotic and especially synbiotic decrease the concentration of triglyceride in prediabetic adults
							*L. acidophilus*				
							*B. bifidum*				
							*B. longum*				

Kassaian, 2020	Iran	80T 40C	52.95	Not reported	Prediabetic subjects	Inulin	*L. acidophilus*	1.5 × 10^9^ CFU	6 mth probiotic synbiotic 6 g/D sachet form	6 mth maltodextrin 6 g/D sachet form	Probiotic effects in prevention and management of obesity and metabolic disorders like diabetes
							*B. bifidum*				
							*B. lactis*				
							*B. longum*				

Mahboobi, 2014	Iran	30T 30C	50.71	76.15	Prediabetic patients	FOS	*L. Casei*	7 × 10^9^	8 wk synbiotic (probiotic + B group vitamins, maltodextrin, lactose, magnesium stearate) 500 mg/D capsule	8 wk starch 500 mg/D capsule	Probiotics did not have significant effects on lipid markers. Positive effects on systolic blood pressure
							*L. acidophilus*	2 × 10^9^			
							*L. rhamnosus*	1.5 × 10^9^			
							*L. bulgaricus*	2 × 10^8^			
							*B. breve*	2 × 10^10^			
							*B. longum*	7 × 10^9^			
							*S. thermophilus*	1.5 × 10^10^			

Sartang, 2018	Iran	44T 43C	45.49	82.8	Overweight and obese patients with metabolic syndrome	FSY	*S. thermophiles*	Not reported	10 wk FSY (contained whey protein, calcium, inulin, and vitamin D) 2 × 250 g/D	10 wk LFY 2 × 250 g/D	Consuming FSY improved body composition and metabolic parameters, while on a calorie-restricted diet
							*L. bulgaricus*	Not reported			
							*B. lactis Bb-12*	10^7^			

Naito, 2017	Japan	50T 50C	47	85.1	Obese prediabetic men	Not reported	*L. casei strain Shirota*	1.0 × 10^11^	8 wk fermented milk form 100 ml bottle/D	8 wk nonfermented milk form 100 ml bottle/D	*L. casei* strain Shirota may favourably affect metabolic abnormalities in obese prediabetic subjects

Rabiei, 2018	Iran	23T 23C	58.95	82.65	Patients with metabolic syndrome	FOS	*L. casei*	2 × 10^8^	12 wk b.i.d synbiotic (probiotic + FOS, magnesium stearate) 500 mg/D capsule	12 wk b.i.d maltodextrin 500 mg/D capsule	Synbiotic improves the status of BMI, FBS, insulin resistance, HOMA-IR, GLP-1, and PYY in patients with metabolic syndrome
							*L. rhamnosus*				
							*S. thermophilus*				
							*B. breve*				
							*L. acidophilus*				
							*B. longum*				
							*L. bulgaricus*				

Rajkumar, 2014	India	15C 30T	20–25 years	BMI (kg/m^2^) 22.43	Healthy young individuals	FOS	*L. salivarius UBL S22*	2 × 10^9^	6 wk probiotic synbiotic 500 mg/D capsule	6 wk gelatin 500 mg/D capsule	Combination of *L*. *salivarius* with FOS more beneficial than *L salivarius* alone

Palacios, 2020	Australia	30T 30C	58.75	100.9	Prediabetic or type 2 diabetic subjects	Cellulose	*L. plantarum*	6 × 10^9^	12 wk f.i.d probiotic + (40 mg microcrystalline cellulose, 5 mg silica, and 10 mg magnesium stearate) capsule	12 wk f.i.d placebo (200 mg microcrystalline cellulose, 10 mg silica, 10 mg magnesium stearate) capsule	Probiotics may act as an adjunctive to metformin by increasing the production of butyrate
							*L. bulgaricus*	3 × 10^9^			
							*L. gasseri*	18 × 10^9^			
							*B. breve*	7.5 × 10^9^			
							*B. animalis sbsp. Lactis*	8 × 10^9^			
							*B. bifidum*	7 × 10^9^			
							*S. thermophilus*	450 × 10^6^			
							*Saccharomyces boulardii*	45 × 10^6^			

Stefanaki, 2019	Greece	16T 16C	14.11	Not reported	Adolescents with prediabetes	Not reported	*S. thermophilus*	450 × 10^9^	4 mth b.i.d (probiotic) counseling to promote a healthy lifestyle + probiotic sachet form	4 mth counseling to promote a healthy lifestyle	Probiotics moderately improve glycemic control and intestinal health
							*B. breve*				
							*B. longum*				
							*B. infantis*				
							*L. acidophilus*				
							*L. plantarum*				
							*L. paracasei*				
							*L. delbreuckii* subspecies bulgaricus				

Tay 2020	New Zealand	17T 16C	52.25	96.56	Prediabetic patients	Not reported	*L. rhamnosus*	6 × 10^9^	12 wk o.d Probiotic Capsule	12 wk o.d placebo (microcrystalline cellulose and dextrose anhydrate) capsule	*L. rhamnosus* probiotic does not have any additional benefits to weight loss or diabetes prevention when combined with an intermittent fasting intervention

Yang, 2015	USA	14T 15C	40.9	80.3	Healthy and prediabetic subjects	XOS	—	—	8 wk XOS 2 g/D capsule	8 wk maltodextrin 2 g/D capsule	XOS may be beneficial in reversing changes in the gut microbiota during the development of diabetes

ELD, elderly adults; MID, mid-age adults; DRS, digestion resistant starch; wk, week; IR, insulin resistance; MetS, metabolic syndrome; mth, month; o.d, once a day; FOS, fructooligosaccharide; FSY, fortified yogurt; LFY, low-fat plain yogurt; b.i.d, two times a day; f.i.d, four times a day; XOS, xylooligosaccharide.

**Table 2:** Lipid parameters changes in different clinical trials assessing the pro-/pre-/synbiotic efficacy on preDM subjects.

No.	Ref	Intervention	TC	LDL-c	HDL-c	TG	VLDL
1	Kassaian et al. [25]	Probiotic & synbiotic	NS^a^	NS	NS	↓	NR^c^
2	Alfa et al. [31]	Prebiotic	NC^b^	NS	NS	NC	**↑**(*P*=0.02)
3	Rabiei et al. [27]	Synbiotic	NC	NC	NC	NS	NR
4	Mahboobi et al. [34]	Probiotic	NS	NS	NS	NS	NR
5	Sartang et al. [23]	Fortified yogurt (FSY) low-fat yogurt (LFY)	NR	**↓**	**↑**	**↓**	NR
6	Tay et al. [22]	Probiotic	NS	NS	NS	NC	NR
7	Rajkumar et al. [30]	Probiotic & prebiotic	**↓**(*P*=0.05)	**↓**(*P*=0.05)	↑NS	**↓**NS	NR
8	Cicero et al. [28]	Probiotic & synbiotic	**↓**(*P* < 0.05)	**↓**(*P* < 0.05)	**↓**(*P* < 0.05)	**↓**(*P* < 0.05)	NR
9	Naito et al. [24]	Probiotic	**↓**(*P*=0.023)	↓*P*=(0.022)	NS	NR	NR

*NS* ^*a*^ *, not significant effect; NC* ^*b*^ *, not changed; NR* ^*c*^ *, not reported*.

## Data Availability

All relevant data are included within the article.

## Supplementary Materials

Supplementary Materials Supplementary data 1. Detailed procedures for the systematic review including its search queries. Supplementary data 2. JBI tool for assessing RCTs.
