# Supplementary material for: The Influence of Probiotics Consumption on Management of Prediabetic State: A Systematic Review of Clinical Trials
Source: Int J Clin Pract. 2022 Sep 12;2022:5963679. doi: 10.1155/2022/5963679 (PMC9484983; doi:10.1155/2022/5963679)
Supplement: Supplementary Materials — Supplementary data 1. Detailed procedures for the systematic review including its search queries. Supplementary data 2. JBI tool for assessing RCTs. [file 5963679.f1.zip › 5963679.f1/Supplementary Data 1.docx]

**Supplementary data1**

**Additional file 1:** Detailed procedures for the systematic review including its search queries.

1. ***Research methodology***

This systematic review was developed based on the PRISMA guidelines (Preferred Reporting Items for Systematic reviews and Meta-analysis), composed of a checklist of 27 items and a four-step flowchart to guide the review.

**
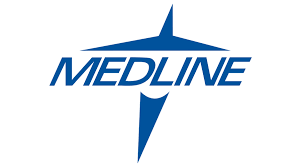
**Searches were made 2010 to 2020 in the scientific databases.

1. **
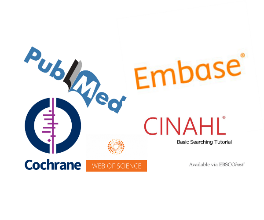
 Medline**, database of the National Library of Medicine, using the following descriptors Medical Subject Headings (MeSH): “prestate diabetes” and “probiotics”
2. **
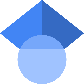
 Embase**, database EMBASE is the European counterpart of MEDLINE and contains over 32 million references
3. **
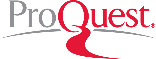
 Google scholor** provides a simple way to broadly search for scholarly literature.
4. **Proquest** is an Ann Arbor, Michigan-based global information-content and technology company, founded in 1938 as University Microfilms by Eugene B. Power. ProQuest provides applications and products for libraries.
5.
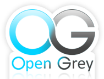

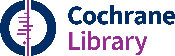
 **The Cochrane Library** (ISSN 1465-1858) is a collection of databases that contain different types of high-quality, independent evidence to inform healthcare decision-making. The Cochrane Library is owned by [Cochrane](http://www.cochrane.org/) and published by [Wiley](http://www.wiley.com/).
6. **Open Grey** provided improved research facilities and the export of records. Notification has been given that as of December 1, 2020 the OpenGrey Repository will be discontinued and preserved as a closed archive.
7. **
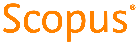
Scopus** is Elsevier’s abstract and citation database launched in 2004. Scopus covers nearly 36,377 titles from approximately 11,678 publishers, of which 34,346 are peer-reviewed journals in top-level subject fields: life sciences, social sciences, physical sciences and health sciences.
8.
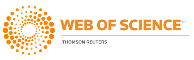
**Web of Science** (previously known as Web of Knowledge) is a website that provides subscription-based access to multiple databases that provide comprehensive citation data for many different [academic disciplines](https://en.wikipedia.org/wiki/Academic_discipline). It was originally produced by the [Institute for Scientific Information](https://en.wikipedia.org/wiki/Institute_for_Scientific_Information) (ISI) and is currently maintained by [Clarivate Analytics](https://en.wikipedia.org/wiki/Clarivate_Analytics) (previously the Intellectual Property and Science business of [Thomson Reuters](https://en.wikipedia.org/wiki/Thomson_Reuters)).
9.
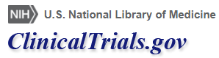
 **clinical trials.gov** is a [registry](https://en.wikipedia.org/wiki/Clinical_trials_registry) of [clinical trials](https://en.wikipedia.org/wiki/Clinical_trial). It is run by the [United States](https://en.wikipedia.org/wiki/United_States) [National Library of Medicine](https://en.wikipedia.org/wiki/National_Library_of_Medicine) (NLM) at the [National Institutes of Health](https://en.wikipedia.org/wiki/National_Institutes_of_Health), and is the largest clinical trials database, holding registrations from over 329,000 trials from 209 countries.
10. **Detailed searches in**

- **Medline**
- ***Free Search***

"prestate diabetes" AND ( "probiotics")

Filters: **Randomized Controlled Trial, Humans**

- ***Indexed Search***
- "prestate diabetes"[Mesh] AND ( "probiotics"[Mesh] )

Filters: **Randomized Controlled Trial, Humans**

- **Embase**
- ***Free* search**

('insulin resistance' OR 'impaired glucose tolerance') AND ('probiotic agent') AND [randomized controlled trial]

- ***Indexed Search***

'probiotic agent'/exp/mj AND ('insulin resistance'/exp/mj OR 'impaired glucose tolerance/exp/mj) AND [randomized controlled trial]/lim AND English:la

- **Google** **scholor**

Prediabetes + probiotics

Return articles dated between 2010-2020

- **Proquest**

("prediabetes") AND (probiotics) AND (“clinical trial*” OR “randomized controlled trials*)

- **The Cochrane Library**

"prediabetes" in Title Abstract Keyword OR "prediabetic state" in Title Abstract Keyword OR "insulin resistance" in Title Abstract Keyword AND "probiotic" in Title Abstract Keyword - (Word variations have been searched)

- **Open** **grey**

Prediabetes AND probiotics

- **Scopus**

TITLE-ABS-KEY ( "prediabetes" )  AND  TITLE-ABS KEY ( "probiotics" )  AND  ( LIMIT-TO ( DOCTYPE ,  "ar"  ) )  AND  ( LIMIT-TO ( LANGUAGE ,  "English" )

- **Web of Science**

TS= ("prediabetes") AND ALL FIELDS= (“probiotic*”) AND ALL FIELDS= (“clinical trial*” OR “randomized controlled trials*)

Timespan: All years.

- **Clinicaltrials.gov**

probiotics | Prediabetic State

OR

probiotics | PreDiabetes

OR

probiotics | Insulin Resistance
