# Supplementary material for: The Influence of Probiotics Consumption on Management of Prediabetic State: A Systematic Review of Clinical Trials
Source: Int J Clin Pract. 2022 Sep 12;2022:5963679. doi: 10.1155/2022/5963679 (PMC9484983; doi:10.1155/2022/5963679)

**Supplementary data**

Table S1: JBI tool for assessing RCTs

| ***Ref*** | ***Q1*** | ***Q2*** | ***Q3*** | ***Q4*** | ***Q5*** | ***Q6*** | ***Q7*** | ***Q8*** | ***Q9*** | ***Q10*** | ***Q11*** | ***Q12*** | ***Q13*** | **Overall appraisal** |
| --- | --- | --- | --- | --- | --- | --- | --- | --- | --- | --- | --- | --- | --- | --- |
| ***Nazila Kassaian 2018*** | *Y* | *Y* | *Y* | *Y* | *Y* | *?* | *Y* | *Y* | *Y* | *Y* | *Y* | *Y* | *Y* | *12* |
| ***Nazila Kassaian 2018*** | *Y* | *Y* | *Y* | *Y* | *Y* | *?* | *Y* | *Y* | *Y* | *Y* | *Y* | *Y* | *Y* | *12* |
| ***Jieping Yang 2015*** | *Y* | *Y* | *N* | *Y* | *Y* | *?* | *Y* | *Y* | *Y* | *Y* | *Y* | *Y* | *N* | *10* |
| ***Charikleia Stefanaki 2019*** | *Y* | *N* | *Y* | *Y* | *N* | *?* | *Y* | *Y* | *Y* | *Y* | *Y* | *Y* | *N* | *9* |
| ***Nazila Kassaian 2020*** | *Y* | *Y* | *Y* | *Y* | *Y* | *?* | *Y* | *Y* | *Y* | *Y* | *Y* | *Y* | *Y* | *12* |
| ***Nazila Kassaian 2019*** | *Y* | *Y* | *Y* | *Y* | *Y* | *?* | *Y* | *Y* | *Y* | *Y* | *Y* | *Y* | *Y* | *12* |
| ***Eiichiro Naito 2017*** | *Y* | *Y* | *Y* | *Y* | *Y* | *?* | *Y* | *Y* | *Y* | *Y* | *Y* | *Y* | *Y* | *12* |
| ***Hemalatha Rajkumar 2014*** | *Y* | *Y* | *Y* | *Y* | *N* | *?* | *Y* | *Y* | *Y* | *Y* | *Y* | *Y* | *N* | *10* |
| ***Mohsen Mohammadi-Sartang 2018*** | *Y* | *Y* | *Y* | *Y* | *Y* | *?* | *Y* | *Y* | *Y* | *Y* | *Y* | *Y* | *Y* | *12* |
| ***Michelle J. Alfa 2018*** | *Y* | *Y* | *Y* | *Y* | *Y* | *?* | *Y* | *Y* | *Y* | *Y* | *Y* | *Y* | *Y* | *12* |
| ***Samira Rabiei 2018*** | *Y* | *Y* | *Y* | *Y* | *Y* | *Y* | *Y* | *Y* | *Y* | *Y* | *Y* | *Y* | *Y* | *13* |
| ***Talia Palacios 2020*** | *Y* | *Y* | *Y* | *Y* | *Y* | *?* | *Y* | *Y* | *Y* | *Y* | *Y* | *Y* | *N* | *11* |
| ***Sepide Mahboobi 2014*** | *Y* | *Y* | *Y* | *Y* | *Y* | *?* | *Y* | *Y* | *Y* | *Y* | *Y* | *Y* | *Y* | *12* |

| ***Arrigo F. G. Cicero 2020*** | *Y* | *Y* | *Y* | *Y* | *Y* | *Y* | *Y* | *Y* | *Y* | *Y* | *Y* | *Y* | *Y* | *13* |
| --- | --- | --- | --- | --- | --- | --- | --- | --- | --- | --- | --- | --- | --- | --- |
| ***Audrey Tay 2020*** | *Y* | *Y* | *Y* | *Y* | *Y* | *?* | *Y* | *Y* | *Y* | *Y* | *Y* | *Y* | *Y* | *12* |

Y= YES, N= NO, UNCLEAR/ NOT APLICAPABLE=?

| **Q1** | **Was true randomization used for assignment of participants to treatment groups?** |
| --- | --- |
| **Q2** | **Was allocation to treatment groups concealed?** |
| **Q3** | **Were treatment groups similar at the baseline?** |
| **Q4** | **Were participants blind to treatment assignment?** |
| **Q5** | **Were those delivering treatment blind to treatment assignment?** |
| **Q6** | **Were outcomes assessors blind to treatment assignment?** |
| **Q7** | **Were treatment groups treated identically other than the intervention of interest?** |
| **Q8** | **Was follow up complete and if not, were differences between groups in terms of their follow up adequately described and analyzed?** |
| **Q9** | **Were participants analyzed in the groups to which they were randomized?** |
| **Q10** | **Were outcomes measured in the same way for treatment groups?** |
| **Q11** | **Were outcomes measured in a reliable way?** |
| **Q12** | **Was appropriate statistical analysis used?** |
| **Q13** | **Was the trial design appropriate, and any deviations from the standard RCT design (individual randomization, parallel groups) accounted for in the conduct and analysis of the trial?** |


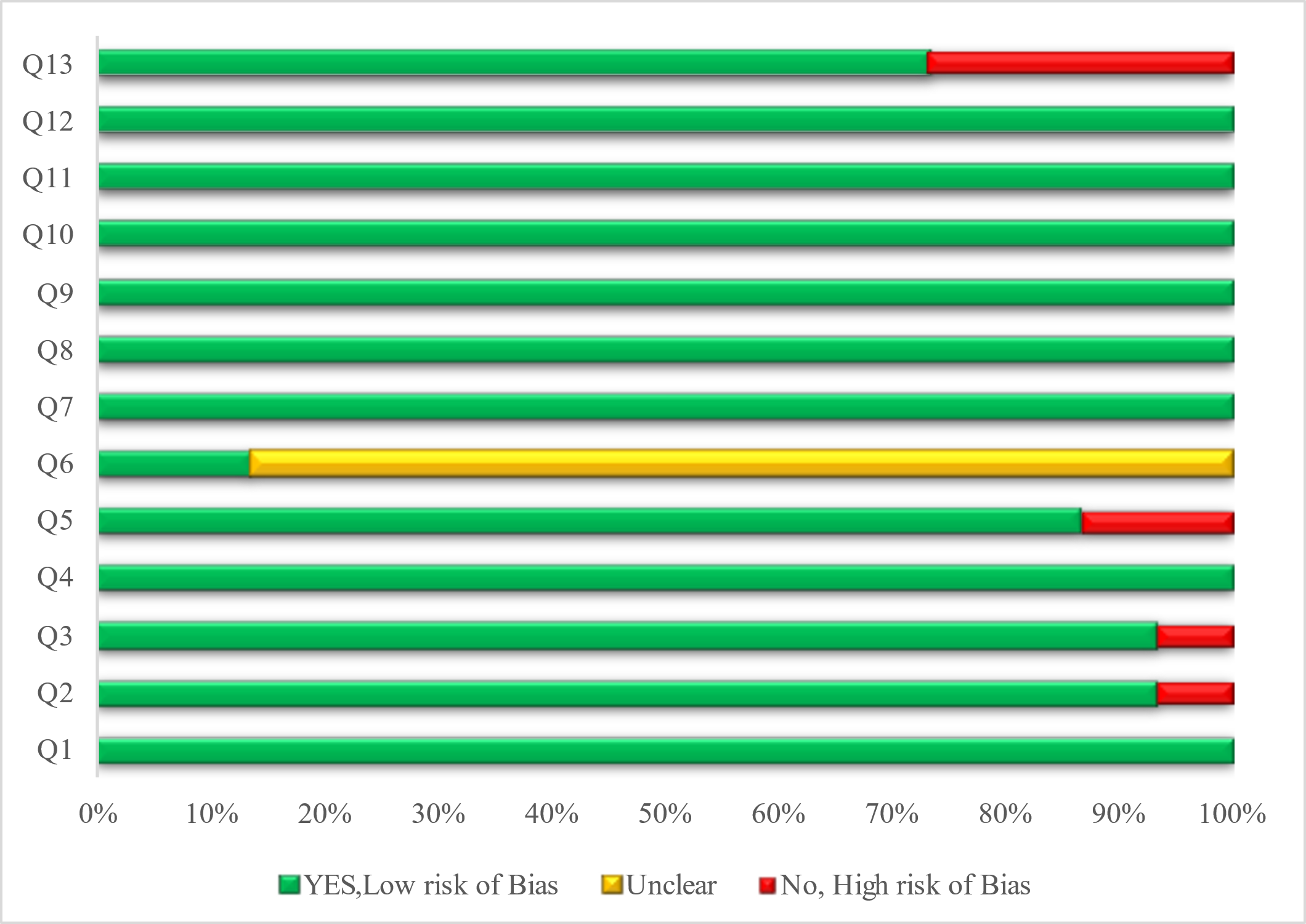

Supplement: Supplementary Materials — Supplementary data 1. Detailed procedures for the systematic review including its search queries. Supplementary data 2. JBI tool for assessing RCTs. [file 5963679.f1.zip › 5963679.f1/SupplementaryData2.docx]
